# Supplementary figures and images for: The association between water intake and future cardiometabolic disease outcomes in the Malmö Diet and Cancer cardiovascular cohort
Source: PLoS One. 2024 Jan 19;19(1):e0296778. doi: 10.1371/journal.pone.0296778 (PMC10798487; doi:10.1371/journal.pone.0296778)

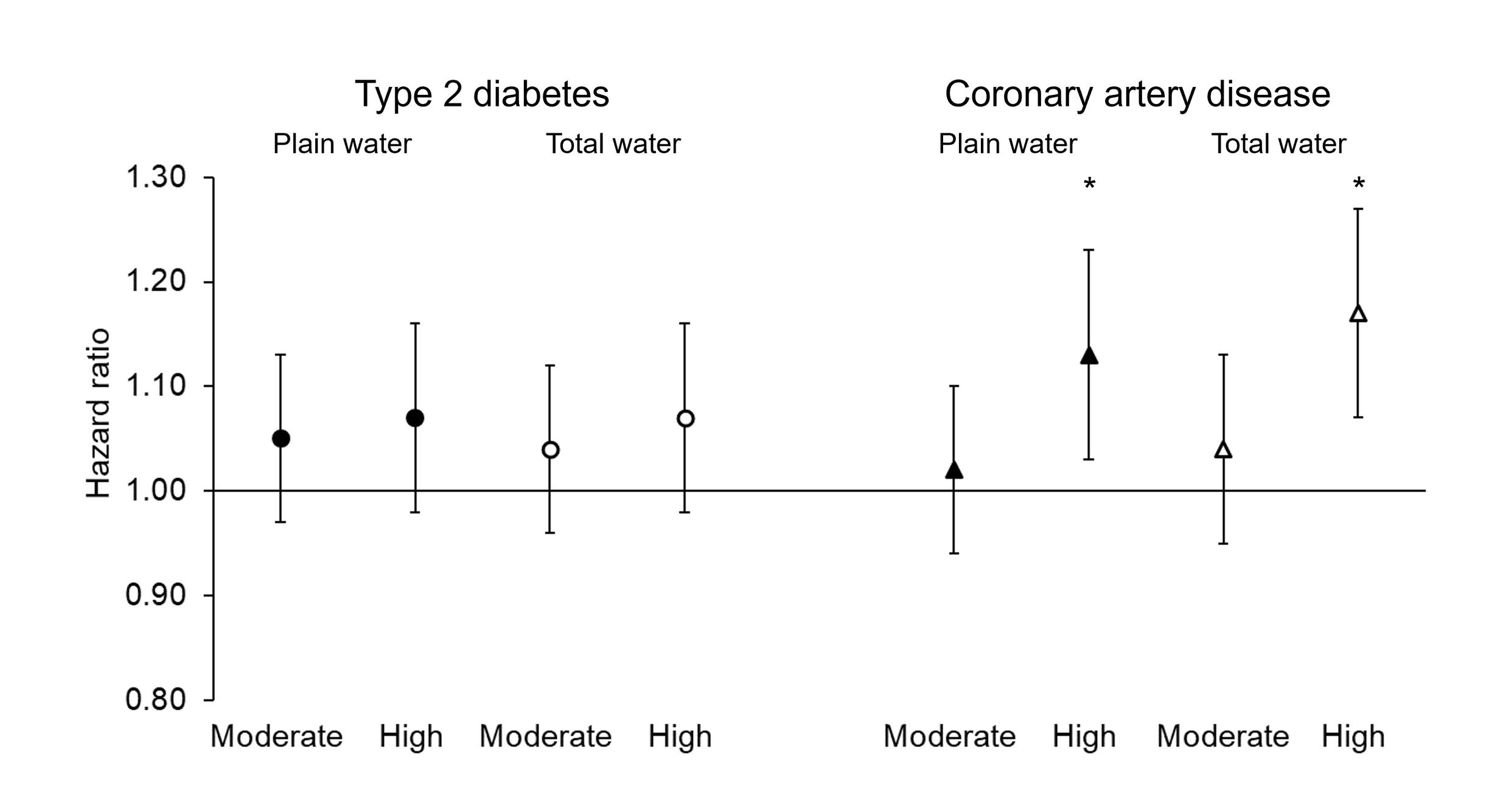

Supplement: S1 Fig — Data represent moderate or high tertiles of intake compared to low intake (reference). Error bars are 95% confidence intervals. * indicates HR is significant (p ≤ 0.05). Black dots (●) indicate plain water and type 2 diabetes; empty dots (○) indicate total water and type 2 diabetes; black triangles (▲) indicate plain water and coronary artery disease; empty triangles (Δ) indicate total water and coronary artery disease. (TIF) [file pone.0296778.s001.tif]
