# Supplementary material for: The association between water intake and future cardiometabolic disease outcomes in the Malmö Diet and Cancer cardiovascular cohort
Source: PLoS One. 2024 Jan 19;19(1):e0296778. doi: 10.1371/journal.pone.0296778 (PMC10798487; doi:10.1371/journal.pone.0296778)
Supplement: S1 Table — (DOCX) [file pone.0296778.s002.docx]

**S1 Table. Sensitivity analyses for type 2 diabetes and coronary artery disease**

|  |  | HR | 95 % CI | *p* | HR | 95 % CI | *p* |
| --- | --- | --- | --- | --- | --- | --- | --- |
|  |  | PLAIN WATER | | | | | |
|  |  | TYPE 2 DIABETES | | | CORONARY ARTERY DISEASE | | |
|  | n | Moderate (*versus* low) intake | | | Moderate (*versus* low) intake | | |
| Main analysis | 25,369 | **1.05** | **0.97, 1.13** | **0.202** | **1.02** | **0.94, 1.10** | **0.662** |
| Continuous model^1^ | 25,369 | 1.02 | 1.00, 1.01 | 0.012 | 1.02 | 1.01, 1.04 | 0.003 |
| Energy adj water^2^ | 25,369 | 1.04 | 0.96, 1.12 | 0.382 | 1.06 | 0.98, 1.14 | 0.181 |
| Energy adj all^3^ | 25,369 | 1.03 | 0.96, 1.12 | 0.392 | 1.06 | 0.98, 1.14 | 0.171 |
| No rep cat adj^4^ | 25,369 | 1.05 | 0.97, 1.13 | 0.202 | 1.02 | 0.94, 1.10 | 0.666 |
| Plausible reporter only | 20,741 | 1.03 | 0.95, 1.12 | 0.521 | 1.05 | 0.96, 1.14 | 0.275 |
| Exclude diet changers | 19,937 | 1.08 | 0.99, 1.17 | 0.084 | 1.04 | 0.95, 1.14 | 0.387 |
| Remove outliers^5^ | 22,832 | 1.07 | 0.99, 1.16 | 0.083 | 0.97 | 0.89, 1.06 | 0.494 |
| No disease in first 2 years | CAD 25,009;  T2D 24,907 | 1.04 | 0.96, 1.13 | 0.305 | 1.01 | 0.93, 1.09 | 0.861 |
| Hypertensive | 15,225 | 1.04 | 0.95, 1.31 | 0.440 | 1.00 | 0.91, 1.10 | 0.971 |
| Normotensive | 10,144 | 1.09 | 0.95, 1.26 | 0.217 | 1.08 | 0.93, 1.27 | 0.319 |
|  |  | High (*versus* low) intake | | | High (*versus* low) intake | | |
| Main analysis | 25,369 | **1.07** | **0.99, 1.16** | **0.075** | **1.13** | **1.04, 1.23** | **0.004** |
| Energy adj water^2^ | 25,369 | 1.10 | 1.01, 1.19 | 0.032 | 1.10 | 1.00, 1.20 | 0.043 |
| Energy adj all^3^ | 25,369 | 1.10 | 1.01, 1.19 | 0.030 | 1.10 | 1.01, 1.20 | 0.035 |
| No rep cat adj^4^ | 25,369 | 1.07 | 0.99, 1.16 | 0.075 | 1.12 | 1.04, 1.23 | 0.004 |
| Plausible reporter only | 20,741 | 1.04 | 0.95, 1.14 | 0.386 | 1.15 | 1.05, 1.26 | 0.002 |
| Exclude diet changers | 19,937 | 1.10 | 1.01, 1.21 | 0.032 | 1.13 | 1.03, 1.25 | 0.010 |
| Remove outliers^5^ | 22,832 | 1.09 | 1.00, 1.18 | 0.045 | 1.10 | 1.01, 1.20 | 0.0.34 |
| No disease in first 2 years | CAD 25,009;  T2D 24,907 | 1.06 | 0.97, 1.14 | 0.139 | 1.13 | 1.03, 1.23 | 0.007 |
| Hypertensive | 15,225 | 1.09 | 0.99, 1.19 | 0.071 | 1.13 | 1.02, 1.24 | 0.015 |
| Normotensive | 10,144 | 1.04 | 0.89, 1.21 | 0.622 | 1.13 | 0.96, 1.13 | 0.146 |
|  |  | TOTAL WATER | | | | | |
|  |  | Moderate (*versus* low) intake | | | Moderate (*versus* low) intake | | |
| Main analysis | 25,369 | **1.04** | **0.96, 1.12** | **0.301** | **1.04** | **0.96 1.13** | **0.335** |
| Continuous model^1^ | 25,369 | 1.01 | 1.00, 1.02 | 0.227 | 1.02 | 1.01, 1.03 | < 0.001 |
| Energy adj water^2^ | 25,369 | 1.02 | 0.95, 1.11 | 0.574 | 1.07 | 0.99, 1.16 | 0.100 |
| Energy adj all^3^ | 25,369 | 1.02 | 0.95, 1.11 | 0.584 | 1.07 | 0.99, 1.16 | 0.093 |
| No rep cat adj^4^ | 25,369 | 1.07 | 0.98, 1.15 | 0.121 | 1.04 | 0.96, 1.13 | 0.339 |
| Plausible reporter only | 20,741 | 1.05 | 0.96, 1.15 | 0.310 | 1.04 | 0.95, 1.13 | 0.428 |
| Exclude diet changers | 19,937 | 1.05 | 0.96, 1.15 | 0.295 | 1.05 | 0.96, 1.15 | 0.276 |
| Remove outliers^5^ | 22,883 | 1.06 | 0.98, 1.16 | 0.159 | 1.03 | 0.94, 1.12 | 0.549 |
| No disease in first 2 years | CAD 25,009;  T2D 24,907 | 1.04 | 0.95, 1.12 | 0.417 | 1.03 | 0.95, 1.12 | 0.478 |
| Hypertensive | 15,225 | 1.07 | 0.98, 1.18 | 0.138 | 1.05 | 0.96, 1.15 | 0.311 |
| Normotensive | 10,144 | 0.04 | 0.89, 1.21 | 0.665 | 1.01 | 0.86, 1.19 | 0.907 |
|  |  | High (*versus* low) intake | | | High (*versus* low) intake | | |
| Main analysis | 25,369 | **1.07** | **0.99, 1.16** | **0.107** | **1.17** | **1.07, 1.27** | **< 0.001** |
| Energy adj water^2^ | 25,369 | 1.02 | 0.94, 1.12 | 0.613 | 1.14 | 1.04, 1.25 | 0.007 |
| Energy adj all^3^ | 25,369 | 1.03 | 0.94, 1.12 | 0.578 | 1.15 | 1.04, 1.26 | 0.005 |
| No rep cat adj^4^ | 25,369 | 1.13 | 1.02, 1.24 | 0.016 | 1.17 | 1.07, 1.27 | < 0.001 |
| Plausible reporter only | 20,741 | 1.09 | 0.97, 1.21 | 0.147 | 1.18 | 1.07, 1.30 | < 0.001 |
| Exclude diet changers | 19,937 | 1.13 | 1.01, 1.26 | 0.037 | 1.15 | 1.04, 1.27 | 0.006 |
| Remove outliers^5^ | 22,883 | 1.11 | 1.00, 1.23 | 0.042 | 1.13 | 1.04, 1.24 | 0.006 |
| No disease in first 2 years | CAD 25,009;  T2D 24,907 | 1.10 | 0.99, 1.21 | 0.066 | 1.17 | 1.07, 1.28 | < 0.001 |
| Hypertensive | 15,225 | 1.14 | 1.01, 1.27 | 0.027 | 1.12 | 1.01, 1.24 | 0.025 |
| Normotensive | 10,144 | 1.10 | 0.91, 1.33 | 0.328 | 1.32 | 1.11, 1.56 | 0.002 |

^1^Continuous model used cups (240 mL) per day as the main predictor variable (rather than tertiles)

^2^Water intakes adjusted per 1000 kcal

^3^All dietary variables (including water) adjusted per 1000 kcal

^4^Not adjusted for energy reporter category

^5^Outliers were defined as those in the top 10 % of fluid intake

All models adjusted for age, sex, diet method, season, smoking, alcohol intake, physical activity level, education, energy intake, energy intake misreporting, BMI, hypertension, lipid lowering medication, apolipoprotein A, apolipoprotein B, processed meat, wholegrains, and for plain water only: non-water beverages. In red are estimates that have changed significance from our original analyses. Abbreviations: CI, confidence interval; HR, hazard ratio; No rep cat adj, not adjusted for reporter category
