# Supplementary material for: The association between water intake and future cardiometabolic disease outcomes in the Malmö Diet and Cancer cardiovascular cohort
Source: PLoS One. 2024 Jan 19;19(1):e0296778. doi: 10.1371/journal.pone.0296778 (PMC10798487; doi:10.1371/journal.pone.0296778)
Supplement: S2 Table — (DOCX) [file pone.0296778.s003.docx]

**S2 Table. Type 2 diabetes analyses with variables that violate proportional hazard assumptions removed**

| Model | HR | 95 % CI | P-value | Model fit (∆ previous model) |
| --- | --- | --- | --- | --- |
|  | Total water | | | |
|  | Moderate (versus low) | | | |
| 1 | 1.03 | 0.96, 1.11 | 0.407 | Χ^2^(2) = 6.781, p = 0.034 |
| 2 | 1.05 | 0.97, 1.13 | 0.249 | Χ^2^(5) = 57.080, p < 0.001 |
| 3 | 1.08 | 0.98, 1.14 | 0.143 | Χ^2^(5) = 162.072, p < 0.001 |
| 4 | 1.07 | 0.99, 1.15 | 0.102 | Χ^2^(6) = 1258.079, p < 0.001 |
| 5 | 1.07 | 0.99, 1.15 | 0.082 | Χ^2^(1) = 15.695, p < 0.001 |
| High (versus low) | | | | |
| 1 | 1.10 | 1.02, 1.19 | 0.011 |  |
| 2 | 1.14 | 1.06, 1.23 | < 0.001 |  |
| 3 | 1.15 | 1.06, 1.24 | < 0.001 |  |
| 4 | 1.15 | 1.06, 1.24 | < 0.001 |  |
| 5 | 1.16 | 1.07, 1.25 | < 0.001 |  |
|  | Plain Water | | | |
|  | Moderate (versus low) | | | |
| 1 | 0.99 | 0.92, 1.06 | 0.748 | Χ^2^(2) = 0.313, p = 0.855 |
| 2 | 0.98 | 0.91, 1.06 | 0.627 | Χ^2^(5) = 51.745, p < 0.001 |
| 3 | 1.00 | 0.93, 1.08 | 0.918 | Χ^2^(5) = 161.749, p < 0.001 |
| 4 | 1.01 | 0.94, 1.09 | 0.748 | Χ^2^(7) = 2171.841, p < 0.001 |
| 5 | 1.02 | 0.94, 1.09 | 0.695 | Χ^2^(2) = 7.192, p = 0.027 |
|  | High (versus low) | | | |
| 1 | 1.01 | 0.94, 1.09 | 0.813 |  |
| 2 | 1.01 | 0.94, 1.09 | 0.716 |  |
| 3 | 1.03 | 0.96, 1.11 | 0.411 |  |
| 4 | 1.01 | 0.94, 1.09 | 0.774 |  |
| 5 | 1.02 | 0.94, 1.10 | 0.684 |  |

Model 1: unadjusted

Model 2: model 1 + age, diet method, season [sex removed]

Model 3: model 2 + smoking, alcohol intake [PAL removed]

Model 4: model 3 + energy intake, energy intake misreporting, hypertension, lipid lowering medication, apolipoprotein A, apolipoprotein B [BMI removed]

Model 5: model 4 + processed meat [wholegrains removed]
