# Supplementary material for: The association between water intake and future cardiometabolic disease outcomes in the Malmö Diet and Cancer cardiovascular cohort
Source: PLoS One. 2024 Jan 19;19(1):e0296778. doi: 10.1371/journal.pone.0296778 (PMC10798487; doi:10.1371/journal.pone.0296778)
Supplement: S3 Table — (DOCX) [file pone.0296778.s004.docx]

**S3 Table. Coronary artery disease analyses with variables that violate proportional hazard assumptions removed**

|  | | HR | 95 % CI | P-value | Model fit (∆ previous model) |
| --- | --- | --- | --- | --- | --- |
| Model | Total water | | | | |
|  | Moderate (versus low) | | | | |
| 1 | | 0.96 | 0.89, 1.04 | 0.318 | Χ^2^(2) = 1.162, p = 0.559 |
| 2 | | 1.02 | 0.94, 1.10 | 0.629 | Χ^2^(5) = 1031.051, p < 0.001 |
| 3 | | 1.03 | 0.95, 1.11 | 0.477 | Χ^2^(8) = 177.586, p < 0.001 |
| 4 | | 0.99 | 0.91, 1.07 | 0.703 | Χ^2^(3) = 533.723, p < 0.001 |
| 5 | | 1.00 | 0.92, 1.08 | 0.937 | Χ^2^(2) = 28.252, p < 0.001 |
| High (versus low) | | | | | |
| 1 | | 0.99 | 0.92, 1.08 | 0.883 |  |
| 2 | | 1.17 | 1.08, 1.27 | < 0.001 |  |
| 3 | | 1.17 | 1.08, 1.27 | < 0.001 |  |
| 4 | | 1.07 | 0.98, 1.16 | 0.122 |  |
| 5 | | 1.10 | 1.01, 1.20 | 0.030 |  |
|  | | Plain water | | | |
|  | | Moderate (versus low) | | | |
| 1 | | 0.89 | 0.83, 0.97 | 0.005 | Χ^2^(2) = 11.869, p = 0.003 |
| 2 | | 0.85 | 0.79, 0.92 | < 0.001 | Χ^2^(5) = 1022.792, p < 0.001 |
| 3 | | 0.88 | 0.81, 0.95 | < 0.001 | Χ^2^(8) = 171.493, p < 0.001 |
| 4 | | 0.92 | 0.85, 1.00 | 0.041 | Χ^2^(3) = 537.586, p < 0.001 |
| 5 | | 0.94 | 0.87, 1.01 | 0.105 | Χ^2^(3) = 30.121, p < 0.001 |
|  | | High (versus low) | | | |
| 1 | | 0.88 | 0.82, 0.96 | 0.002 |  |
| 2 | | 0.86 | 0.78, 0.92 | < 0.001 |  |
| 3 | | 0.90 | 0.82, 0.97 | 0.005 |  |
| 4 | | 0.98 | 0.91, 1.06 | 0.632 |  |
| 5 | | 1.01 | 0.93, 1.09 | 0.836 |  |

Model 1: unadjusted

Model 2: model 1 + age, diet method, season [sex removed]

Model 3: model 2 + alcohol intake, physical activity level, education [smoking removed]

Model 4: model 3 + energy intake, energy intake misreporting, apolipoprotein A [BMI, hypertension, lipid lowering medication, ApoB removed]

Model 5: model 4 + processed meat, wholegrains
