# Supplementary material for: The association between water intake and future cardiometabolic disease outcomes in the Malmö Diet and Cancer cardiovascular cohort
Source: PLoS One. 2024 Jan 19;19(1):e0296778. doi: 10.1371/journal.pone.0296778 (PMC10798487; doi:10.1371/journal.pone.0296778)
Supplement: S4 Table — (DOCX) [file pone.0296778.s005.docx]

**S4 Table. Type 2 diabetes analyses stratified by variables that violate proportional hazard assumptions**

|  | HR | 95 % CI | *p* | HR | 95 % CI | *p* |
| --- | --- | --- | --- | --- | --- | --- |
|  | Plain water | | | Total water | | |
|  | Moderate (*versus* low) intake | | | Moderate (*versus* low) intake | | |
| BMI low | 0.97 | 0.84, 1.11 | 0.631 | 1.09 | 0.95, 1.26 | 0.219 |
| BMI high | 1.10 | 1.01, 1.21 | 0.031 | 1.10 | 0.94, 1.28 | 0.241 |
| Age low | 1.06 | 0.96, 1.18 | 0.264 | 1.05 | 0.94, 1.17 | 0.422 |
| Age high | 1.05 | 0.941, 1.17 | 0.395 | 1.03 | 0.93, 1.15 | 0.554 |
| WG low | 1.10 | 1.00, 1.22 | 0.063 | 1,05 | 0.95, 1.16 | 0.368 |
| WG high | 1.00 | 0.89, 1.12 | 0.989 | 1.04 | 0.92, 1.16 | 0.553 |
| PAL 1 | 0.98 | 0.84, 1.13 | 0.736 | 1.10 | 0.95, 1.28 | 0.193 |
| PAL 2 | 1.19 | 1.01, 1.41 | 0.041 | 1.00 | 0.84, 1.18 | 0.951 |
| PAL 3 | 1.06 | 0.88, 1.26 | 0.553 | 1.01 | 0.85, 1.20 | 0.925 |
| PAL 4 | 1.00 | 0.83, 1.19 | 0.973 | 1.06 | 0.88, 1.28 | 0.555 |
| PAL 5 | 1.05 | 0.88, 1.25 | 0.608 | 1.03 | 0.86, 1.24 | 0.741 |
| Original model | **1.05** | **0.97, 1.13** | **0.202** | **1.04** | **0.96, 1.12** | **0.301** |
|  | High (*versus* low) intake | | | High (*versus* low) intake | | |
| BMI low | 1.00 | 0.86, 1.16 | 0.995 | 1.05 | 0.96, 1.15 | 0.301 |
| BMI high | 1.17 | 1.06, 1.28 | 0.001 | 1.15 | 1.04, 1.26 | 0.005 |
| Age low | 1.09 | 0.98, 1.21 | 0.128 | 1.06 | 0.94, 1.18 | 0.355 |
| Age high | 1.06 | 0.95, 1.19 | 0.320 | 1.08 | 0.96, 1.21 | 0.205 |
| WG low | 1.09 | 0.98, 1.22 | 0.111 | 1.12 | 0.01, 1.26 | 0.039 |
| WG high | 1.05 | 0.94, 1.12 | 0.396 | 1.02 | 0.91, 1.15 | 0.714 |
| PAL 1 | 1.19 | 1.02, 1.39 | 0.025 | 1.17 | 1.00, 1.38 | 0.050 |
| PAL 2 | 0.99 | 0.98, 1.01 | 0.925 | 0.98 | 0.82, 1.18 | 0.864 |
| PAL 3 | 1.11 | 0.93, 1.34 | 0.254 | 1.05 | 0.87, 1.28 | 0.591 |
| PAL 4 | 1.03 | 0.85, 1.24 | 0.780 | 1.13 | 0.93, 1.14 | 0.209 |
| PAL 5 | 1.03 | 0.86, 1.23 | 0.748 | 0.99 | 0.82, 1.19 | 0.873 |
| Original model | **1.07** | **0.99, 1.16** | **0.075** | **1.07** | **0.99, 1.16** | **0.107** |

Abbreviations: BMI, body mass index; PAL, physical activity level (quintiles), WG, wholegrain intake

Median split was used to stratify continuous variables. Medians (interquartile ranges) were as follows for the relevant variables: BMI 22.9 (21.5, 24.0) and 27.8 (26.3, 29.1) kg/m^2^; age 51 (49, 54) and 64 (61, 67) years; wholegrain intake 0.26 (0.06, 0.46) and 1.38 (0.99, 2.00) portions/day
