# Supplementary material for: The association between water intake and future cardiometabolic disease outcomes in the Malmö Diet and Cancer cardiovascular cohort
Source: PLoS One. 2024 Jan 19;19(1):e0296778. doi: 10.1371/journal.pone.0296778 (PMC10798487; doi:10.1371/journal.pone.0296778)
Supplement: S5 Table — (DOCX) [file pone.0296778.s006.docx]

**S5 Table. Coronary artery disease analyses stratified by variables that violate proportional hazard assumptions**

|  | HR | 95 % CI | *p* | HR | 95 % CI | *p* |
| --- | --- | --- | --- | --- | --- | --- |
|  | Plain water | | | Total water | | |
|  | Moderate (*versus* low) intake | | | Moderate (*versus* low) intake | | |
| Male | 1.05 | 0.95, 1.16 | 0.360 | 1.00 | 0.90, 1.11 | 0.938 |
| Female | 0.97 | 0.85, 1.10 | 0.613 | 1.11 | 0.98, 1.25 | 0.109 |
| Non-smoker | 1.02 | 0.93, 1.13 | 0.682 | 1.04 | 0.94, 1.15 | 0.451 |
| Smoker | 1.01 | 0.88, 1.16 | 0.858 | 1.04 | 0.90, 1.20 | 0.619 |
| BMI low | 1.03 | 0.91, 1.16 | 0.674 | 1.03 | 0.91, 1.17 | 0.601 |
| BMI high | 1.02 | 0.92, 1.13 | 0.722 | 1.06 | 0.95, 1.18 | 0.295 |
| Hypertension | 1.08 | 0.93, 1.27 | 0.319 | 1.01 | 0.86, 1.19 | 0.903 |
| Normotension | 1.00 | 0.91, 1.10 | 0.973 | 1.05 | 0.96, 1.15 | 0.309 |
| No medication | 1.01 | 0.93, 1.10 | 0.811 | 1.05 | 0.96, 1.14 | 0.299 |
| Medication use | 1.17 | 0.77, 1.77 | 0.457 | 1.02 | 0.68, 1.54 | 0.934 |
| ApoB low | 1.03 | 0.90, 1.18 | 0.690 | 1.04 | 0.91, 1.20 | 0.556 |
| ApoB high | 1.02 | 0.92, 1.12 | 0.728 | 1.03 | 0.93, 1.13 | 0.629 |
| Original model | **1.02** | **0.94, 1.10** | **0.662** | **1.04** | **0.96 1.13** | **0.335** |
|  | High (*versus* low) intake | | | High (*versus* low) intake | | |
| Male | 1.14 | 1.02, 1.27 | 0.017 | 1.12 | 1.00, 1.26 | 0.052 |
| Female | 1.10 | 0.97, 1.26 | 0.152 | 1.25 | 1.10, 1.43 | < 0.001 |
| Non-smoker | 1.15 | 1.04, 1.27 | 0.007 | 1.15 | 1.04, 1.28 | 0.009 |
| Smoker | 1.10 | 0.95, 1.27 | 0.212 | 1.19 | 1.03, 1.38 | 0.021 |
| BMI low | 1.12 | 0.98, 1.27 | 0.101 | 1.16 | 1.01, 1.32 | 0.035 |
| BMI high | 1.15 | 1.04, 1.28 | 0.008 | 1.20 | 1.07, 1.34 | 0.001 |
| Hypertension | 1.13 | 0.96, 1.34 | 0.146 | 1.32 | 1.11, 1.56 | 0.002 |
| Normotension | 1.13 | 1.02, 1.24 | 0.015 | 1.12 | 1.01, 1.24 | 0.025 |
| No medication | 1.13 | 1.03, 1.22 | 0.006 | 1.18 | 1.08, 1.29 | < 0.001 |
| Medication use | 1.24 | 0.81, 1.90 | 0.326 | 0.98 | 0.63, 1.52 | 0.920 |
| ApoB low | 1.18 | 1.02, 1.35 | 0.025 | 1.17 | 1.01, 1.36 | 0.033 |
| ApoB high | 1.10 | 0.99, 1.22 | 0.077 | 1.14 | 1.03, 1.27 | 0.015 |
| Original model | **1.13** | **1.04, 1.23** | **0.004** | **1.17** | **1.07, 1.27** | **< 0.001** |

Abbreviations: ApoB, apolipoprotein B; BMI, body mass index

Median split was used to stratify continuous variables. Medians (interquartile ranges) were as follows for the relevant variables: BMI 22.9 (21.5, 24.0) and 27.8 (26.3, 29.1) kg/m^2^; apolipoprotein B : 89 (78, 97) and 123 (114, 137) mg/dL
