# Supplementary material for: The association between water intake and future cardiometabolic disease outcomes in the Malmö Diet and Cancer cardiovascular cohort
Source: PLoS One. 2024 Jan 19;19(1):e0296778. doi: 10.1371/journal.pone.0296778 (PMC10798487; doi:10.1371/journal.pone.0296778)
Supplement: Attachment — Submitted filename: Response to reviewers.docx [file pone.0296778.s007.docx]

Dear Editor,

Thank you for organising review of our manuscript and allowing for more time for us to address the comments. We have addressed each reviewer comment below (our responses in blue), and submitted a clean and tracked changes version of our updated manuscript.

## Reviewer comments

Reviewer #1 comment: This study provided a longitudinal analysis on the association between baseline water intake and incidence of CAD and type 2 diabetes among a large cohort using cox proportional hazards models. This study adds to the existing literature on hydration and health, with results suggesting an association between water and CAD risk (opposite to the expected relationship) but not type 2 diabetes risk in this cohort. I have a few suggestions which may enhance the quality of this paper.

• The cox proportional hazards analyses include quite a lot of covariates for this type of model despite the large sample size. Can you please provide the concordance statistics as some indication of model fit changes with the inclusion or omission of some of these variables?

Author response: Thank you for these thoughtful comments. For ease, we have summarised model fit statistics below:

**Change from previous model:**

| Model | Total water | | Plain water | |
| --- | --- | --- | --- | --- |
|  | Type 2 diabetes | CAD | Type 2 diabetes | CAD |
| 1 | Χ^2^(2) = 6.781,  p = 0.034 | Χ^2^(2) = 1.162,  p = 0.559 | Χ^2^(2) = 313,  p = 0.855 | Χ^2^(2) = 11.869,  p = 0.003 |
| 2 | Χ^2^(6) = 265.684,  p < 0.001 | Χ^2^(6) = 1846.270, p < 0.001 | Χ^2^(6) = 276.215,  p < 0.001 | Χ^2^(6) = 1832.507,  p < 0.001 |
| 3 | Χ^2^(9) = 231.842,  p < 0.001 | Χ^2^(9) = 312.649,  p < 0.001 | Χ^2^(9) = 226.870,  p < 0.001 | Χ^2^(9) = 313.841,  p < 0.001 |
| 4 | Χ^2^(7) = 1934.622,  p < 0.001 | Χ^2^(7) = 605.662,  p < 0.001 | Χ^2^(7) = 1936.042,  p < 0.001 | Χ^2^(7) = 603.319,  p < 0.001 |
| 5 | Χ^2^(2) = 7.546,  p = 0.023 | Χ^2^(2) = 4.214,  p = 0.122 | Χ^2^(3) = 8.212,  p = 0.042 | Χ^2^(3) = 8.392,  p = 0.039 |

**Overall model:**

| Model | Total water | | Plain water | |
| --- | --- | --- | --- | --- |
|  | Type 2 diabetes | CAD | Type 2 diabetes | CAD |
| 1 | Χ^2^(2) = 6.816,  p = 0.033 | Χ^2^(2) = 1.158,  p = 0.561 | Χ^2^(2) = 0.313,  p = 0.855 | Χ^2^(2) = 12.017,  p = 0.002 |
| 2 | Χ^2^(8) = 280.983,  p < 0.001 | Χ^2^(8) = 1895.698, p < 0.001 | Χ^2^(8) = 285.220,  p < 0.001 | Χ^2^(8) = 1893.881,  p < 0.001 |
| 3 | Χ^2^(17) = 515.794,  p < 0.001 | Χ^2^(17) = 2180.547, p < 0.001 | Χ^2^(17) = 514.364,  p < 0.001 | Χ^2^(17) = 2180.901,  p < 0.001 |
| 4 | Χ^2^(24) = 2603.444,  p < 0.001 | Χ^2^(24) = 2699.212, p < 0.001 | Χ^2^(24) = 2604.029,  p < 0.001 | Χ^2^(24) = 2695.973,  p < 0.001 |
| 5 | Χ^2^(26) = 2609.835,  p < 0.001 | Χ^2^(26) = 2703.905, p < 0.001 | Χ^2^(27) = 2611.414,  p < 0.001 | Χ^2^(27) = 2703.386,  p < 0.001 |

As can be seen, nearly all models have a significant fit, and nearly all models add significantly to the previous model.

We have also re-run the model with the omission of some variables, taking into account your further comments (please see below, as your further comments helped us determine which variables to omit).

Reviewer #1 comment: Additionally, given the complexity with these additional variables, can you confirm if the models met the proportional hazards assumptions for each covariate?

Author response: The following did not meet the proportional hazards assumptions:

| CAD models | | T2D models | |
| --- | --- | --- | --- |
| Variable name | P-value | Variable name | P-value |
| Sex | < 0.001 | Age | < 0.001 |
| Smoking | < 0.001 | PAL | ≤ 0.005 |
| BMI | ≤ 0.045 | BMI | < 0.001 |
| Hypertension | 0.004 | Wholegrain intake | ≤ 0.011 |
| Lipid lowering medication | 0.001 |  |  |
| ApoB | ≤ 0.007 |  |  |

As per your suggestion above, we have used these as a basis to test the results with the omission of some variables, with results (including fit statistics) below. Results that change significance compared to our original analyses are in red.

**Type 2 diabetes**

| Model | HR | 95 % CI | P-value | Model fit (∆ previous model) |
| --- | --- | --- | --- | --- |
|  | Total water | | | |
|  | Moderate (versus low) | | | |
| 1 | 1.03 | 0.96, 1.11 | 0.407 | Χ^2^(2) = 6.781, p = 0.034 |
| 2 | 1.05 | 0.97, 1.13 | 0.249 | Χ^2^(5) = 57.080, p < 0.001 |
| 3 | 1.08 | 0.98, 1.14 | 0.143 | Χ^2^(5) = 162.072, p < 0.001 |
| 4 | 1.07 | 0.99, 1.15 | 0.102 | Χ^2^(6) = 1258.079, p < 0.001 |
| 5 | 1.07 | 0.99, 1.15 | 0.082 | Χ^2^(1) = 15.695, p < 0.001 |
| High (versus low) | | | | |
| 1 | 1.10 | 1.02, 1.19 | 0.011 |  |
| 2 | 1.14 | 1.06, 1.23 | < 0.001 |  |
| 3 | 1.15 | 1.06, 1.24 | < 0.001 |  |
| 4 | 1.15 | 1.06, 1.24 | < 0.001 |  |
| 5 | 1.16 | 1.07, 1.25 | < 0.001 |  |
|  | Plain Water | | | |
|  | Moderate (versus low) | | | |
| 1 | 0.99 | 0.92, 1.06 | 0.748 | Χ^2^(2) = 0.313, p = 0.855 |
| 2 | 0.98 | 0.91, 1.06 | 0.627 | Χ^2^(5) = 51.745, p < 0.001 |
| 3 | 1.00 | 0.93, 1.08 | 0.918 | Χ^2^(5) = 161.749, p < 0.001 |
| 4 | 1.01 | 0.94, 1.09 | 0.748 | Χ^2^(7) = 2171.841, p < 0.001 |
| 5 | 1.02 | 0.94, 1.09 | 0.695 | Χ^2^(2) = 7.192, p = 0.027 |
|  | High (versus low) | | | |
| 1 | 1.01 | 0.94, 1.09 | 0.813 |  |
| 2 | 1.01 | 0.94, 1.09 | 0.716 |  |
| 3 | 1.03 | 0.96, 1.11 | 0.411 |  |
| 4 | 1.01 | 0.94, 1.09 | 0.774 |  |
| 5 | 1.02 | 0.94, 1.10 | 0.684 |  |

Model 1: unadjusted

Model 2: model 1 + age, diet method, season [sex removed]

Model 3: model 2 + smoking, alcohol intake [PAL removed]

Model 4: model 3 + energy intake, energy intake misreporting, hypertension, lipid lowering medication, apolipoprotein A, apolipoprotein B [BMI removed]

Model 5: model 4 + processed meat [wholegrains removed]

**Coronary artery disease**

|  | | HR | 95 % CI | P-value | Model fit (∆ previous model) |
| --- | --- | --- | --- | --- | --- |
| Model | Total water | | | | |
|  | Moderate (versus low) | | | | |
| 1 | | 0.96 | 0.89, 1.04 | 0.318 | Χ^2^(2) = 1.162, p = 0.559 |
| 2 | | 1.02 | 0.94, 1.10 | 0.629 | Χ^2^(5) = 1031.051, p < 0.001 |
| 3 | | 1.03 | 0.95, 1.11 | 0.477 | Χ^2^(8) = 177.586, p < 0.001 |
| 4 | | 0.99 | 0.91, 1.07 | 0.703 | Χ^2^(3) = 533.723, p < 0.001 |
| 5 | | 1.00 | 0.92, 1.08 | 0.937 | Χ^2^(2) = 28.252, p < 0.001 |
| High (versus low) | | | | | |
| 1 | | 0.99 | 0.92, 1.08 | 0.883 |  |
| 2 | | 1.17 | 1.08, 1.27 | < 0.001 |  |
| 3 | | 1.17 | 1.08, 1.27 | < 0.001 |  |
| 4 | | 1.07 | 0.98, 1.16 | 0.122 |  |
| 5 | | 1.10 | 1.01, 1.20 | 0.030 |  |
|  | | Plain water | | | |
|  | | Moderate (versus low) | | | |
| 1 | | 0.89 | 0.83, 0.97 | 0.005 | Χ^2^(2) = 11.869, p = 0.003 |
| 2 | | 0.85 | 0.79, 0.92 | < 0.001 | Χ^2^(5) = 1022.792, p < 0.001 |
| 3 | | 0.88 | 0.81, 0.95 | < 0.001 | Χ^2^(8) = 171.493, p < 0.001 |
| 4 | | 0.92 | 0.85, 1.00 | 0.041 | Χ^2^(3) = 537.586, p < 0.001 |
| 5 | | 0.94 | 0.87, 1.01 | 0.105 | Χ^2^(3) = 30.121, p < 0.001 |
|  | | High (versus low) | | | |
| 1 | | 0.88 | 0.82, 0.96 | 0.002 |  |
| 2 | | 0.86 | 0.78, 0.92 | < 0.001 |  |
| 3 | | 0.90 | 0.82, 0.97 | 0.005 |  |
| 4 | | 0.98 | 0.91, 1.06 | 0.632 |  |
| 5 | | 1.01 | 0.93, 1.09 | 0.836 |  |

Model 1: unadjusted

Model 2: model 1 + age, diet method, season [sex removed]

Model 3: model 2 + alcohol intake, physical activity level, education [smoking removed]

Model 4: model 3 + energy intake, energy intake misreporting, apolipoprotein A [BMI, hypertension, lipid lowering medication, ApoB removed]

Model 5: model 4 + processed meat, wholegrains

To further assess the impact of these (as results are liable to change simply from removing variables), we stratified the analysis by the offending variables, with results below (differences to the original model in red).

**Type 2 diabetes**

|  | HR | 95 % CI | *p* | HR | 95 % CI | *p* |
| --- | --- | --- | --- | --- | --- | --- |
|  | Plain water | | | Total water | | |
|  | Moderate (*versus* low) intake | | | Moderate (*versus* low) intake | | |
| BMI low | 0.97 | 0.84, 1.11 | 0.631 | 1.09 | 0.95, 1.26 | 0.219 |
| BMI high | 1.10 | 1.01, 1.21 | 0.031 | 1.10 | 0.94, 1.28 | 0.241 |
| Age low | 1.06 | 0.96, 1.18 | 0.264 | 1.05 | 0.94, 1.17 | 0.422 |
| Age high | 1.05 | 0.941, 1.17 | 0.395 | 1.03 | 0.93, 1.15 | 0.554 |
| WG low | 1.10 | 1.00, 1.22 | 0.063 | 1,05 | 0.95, 1.16 | 0.368 |
| WG high | 1.00 | 0.89, 1.12 | 0.989 | 1.04 | 0.92, 1.16 | 0.553 |
| PAL 1 | 0.98 | 0.84, 1.13 | 0.736 | 1.10 | 0.95, 1.28 | 0.193 |
| PAL 2 | 1.19 | 1.01, 1.41 | 0.041 | 1.00 | 0.84, 1.18 | 0.951 |
| PAL 3 | 1.06 | 0.88, 1.26 | 0.553 | 1.01 | 0.85, 1.20 | 0.925 |
| PAL 4 | 1.00 | 0.83, 1.19 | 0.973 | 1.06 | 0.88, 1.28 | 0.555 |
| PAL 5 | 1.05 | 0.88, 1.25 | 0.608 | 1.03 | 0.86, 1.24 | 0.741 |
| Original model | **1.05** | **0.97, 1.13** | **0.202** | **1.04** | **0.96, 1.12** | **0.301** |
|  | High (*versus* low) intake | | | High (*versus* low) intake | | |
| BMI low | 1.00 | 0.86, 1.16 | 0.995 | 1.05 | 0.96, 1.15 | 0.301 |
| BMI high | 1.17 | 1.06, 1.28 | 0.001 | 1.15 | 1.04, 1.26 | 0.005 |
| Age low | 1.09 | 0.98, 1.21 | 0.128 | 1.06 | 0.94, 1.18 | 0.355 |
| Age high | 1.06 | 0.95, 1.19 | 0.320 | 1.08 | 0.96, 1.21 | 0.205 |
| WG low | 1.09 | 0.98, 1.22 | 0.111 | 1.12 | 0.01, 1.26 | 0.039 |
| WG high | 1.05 | 0.94, 1.12 | 0.396 | 1.02 | 0.91, 1.15 | 0.714 |
| PAL 1 | 1.19 | 1.02, 1.39 | 0.025 | 1.17 | 1.00, 1.38 | 0.050 |
| PAL 2 | 0.99 | 0.98, 1.01 | 0.925 | 0.98 | 0.82, 1.18 | 0.864 |
| PAL 3 | 1.11 | 0.93, 1.34 | 0.254 | 1.05 | 0.87, 1.28 | 0.591 |
| PAL 4 | 1.03 | 0.85, 1.24 | 0.780 | 1.13 | 0.93, 1.14 | 0.209 |
| PAL 5 | 1.03 | 0.86, 1.23 | 0.748 | 0.99 | 0.82, 1.19 | 0.873 |
| Original model | **1.07** | **0.99, 1.16** | **0.075** | **1.07** | **0.99, 1.16** | **0.107** |

Abbreviations: BMI, body mass index; PAL, physical activity level (quintiles), WG, wholegrain intake

Median split was used to stratify continuous variables. Medians (interquartile ranges) were as follows for the relevant variables: BMI 22.9 (21.5, 24.0) and 27.8 (26.3, 29.1) kg/m^2^; age 51 (49, 54) and 64 (61, 67) years; wholegrain intake 0.26 (0.06, 0.46) and 1.38 (0.99, 2.00) portions/day

**Coronary artery disease**

|  | HR | 95 % CI | *p* | HR | 95 % CI | *p* |
| --- | --- | --- | --- | --- | --- | --- |
|  | Plain water | | | Total water | | |
|  | Moderate (*versus* low) intake | | | Moderate (*versus* low) intake | | |
| Male | 1.05 | 0.95, 1.16 | 0.360 | 1.00 | 0.90, 1.11 | 0.938 |
| Female | 0.97 | 0.85, 1.10 | 0.613 | 1.11 | 0.98, 1.25 | 0.109 |
| Non-smoker | 1.02 | 0.93, 1.13 | 0.682 | 1.04 | 0.94, 1.15 | 0.451 |
| Smoker | 1.01 | 0.88, 1.16 | 0.858 | 1.04 | 0.90, 1.20 | 0.619 |
| BMI low | 1.03 | 0.91, 1.16 | 0.674 | 1.03 | 0.91, 1.17 | 0.601 |
| BMI high | 1.02 | 0.92, 1.13 | 0.722 | 1.06 | 0.95, 1.18 | 0.295 |
| Hypertension | 1.08 | 0.93, 1.27 | 0.319 | 1.01 | 0.86, 1.19 | 0.903 |
| Normotension | 1.00 | 0.91, 1.10 | 0.973 | 1.05 | 0.96, 1.15 | 0.309 |
| No medication | 1.01 | 0.93, 1.10 | 0.811 | 1.05 | 0.96, 1.14 | 0.299 |
| Medication use | 1.17 | 0.77, 1.77 | 0.457 | 1.02 | 0.68, 1.54 | 0.934 |
| ApoB low | 1.03 | 0.90, 1.18 | 0.690 | 1.04 | 0.91, 1.20 | 0.556 |
| ApoB high | 1.02 | 0.92, 1.12 | 0.728 | 1.03 | 0.93, 1.13 | 0.629 |
| Original model | **0.99** | **0.93, 1.08** | **0.993** | **1.04** | **0.96 1.13** | **0.335** |
|  | High (*versus* low) intake | | | High (*versus* low) intake | | |
| Male | 1.14 | 1.02, 1.27 | 0.017 | 1.12 | 1.00, 1.26 | 0.052 |
| Female | 1.10 | 0.97, 1.26 | 0.152 | 1.25 | 1.10, 1.43 | < 0.001 |
| Non-smoker | 1.15 | 1.04, 1.27 | 0.007 | 1.15 | 1.04, 1.28 | 0.009 |
| Smoker | 1.10 | 0.95, 1.27 | 0.212 | 1.19 | 1.03, 1.38 | 0.021 |
| BMI low | 1.12 | 0.98, 1.27 | 0.101 | 1.16 | 1.01, 1.32 | 0.035 |
| BMI high | 1.15 | 1.04, 1.28 | 0.008 | 1.20 | 1.07, 1.34 | 0.001 |
| Hypertension | 1.13 | 0.96, 1.34 | 0.146 | 1.32 | 1.11, 1.56 | 0.002 |
| Normotension | 1.13 | 1.02, 1.24 | 0.015 | 1.12 | 1.01, 1.24 | 0.025 |
| No medication | 1.13 | 1.03, 1.22 | 0.006 | 1.18 | 1.08, 1.29 | < 0.001 |
| Medication use | 1.24 | 0.81, 1.90 | 0.326 | 0.98 | 0.63, 1.52 | 0.920 |
| ApoB low | 1.18 | 1.02, 1.35 | 0.025 | 1.17 | 1.01, 1.36 | 0.033 |
| ApoB high | 1.10 | 0.99, 1.22 | 0.077 | 1.14 | 1.03, 1.27 | 0.015 |
| Original model | **1.12** | **1.03, 1.21** | **0.008** | **1.17** | **1.07, 1.27** | **< 0.001** |

Abbreviations: ApoB, apolipoprotein B; BMI, body mass index

Median split was used to stratify continuous variables. Medians (interquartile ranges) were as follows for the relevant variables: BMI 22.9 (21.5, 24.0) and 27.8 (26.3, 29.1) kg/m^2^; apolipoprotein B : 89 (78, 97) and 123 (114, 137) mg/dL

As seen above, the following led to different results compared to the original model:

**T2D**

- BMI high (both results for plain water; high vs low comparison for total water)
- Age – similar results to our original analyses for all comparisons
- Wholegrain (high vs low total water)
- PAL1 (high vs low plain water)
- PAL2 (moderate vs low plain water)

**CAD**

- Sex (high vs low plain water; high vs low total water)
- Smoking (high vs low plain water)
- BMI – similar results to our original analyses for all comparisons
- Hypertension (high vs low plain water)
- Lipid lowering medication (high vs low plain water)
- ApoB (high vs low plain water)

We have reported these in the manuscript (see text below) and included them in the Supplementary Material (S2 and S3 Tables).

In the methods we have included the following:

“All variables were checked for the proportional hazards assumption by including a time-dependent covariate. Some variables violated this assumption. To test their impact, we re-ran the analyses omitting these variables, and also stratified the analyses by these variables (pre-defined categories or median split).” (lines 177-181)

In the results we included the following text:

“Removing and/or stratifying by variables that violated proportional hazard assumptions yielded largely similar results, though some deviations were noted (S2-S5 Tables).” (lines 279-281)

In the discussion we included the following text:

“We also found some assumption violations. Removing these variables from our model yielded some differences in results (S2 and S3 Tables, but it is unclear whether this was due to controlling for fewer variables or removing violations. To partially overcome this, we stratified our analyses by the offending variables, yielding largely similar results, though some differences were found (S4 and S5 Tables). Therefore some caution is required when interpreting our findings, and we emphasise the need for replication alongside mechanistic research.” (lines 412-418)

Reviewer #1 comment: Can you provide more rationale for the covariates used in the models?

Author response: We were concerned about the multitude of influences regarding water intake, as well as some methodological factors (hence the inclusion of dietary collection method and season). A rundown of reasoning is as follows:

- Age and sex both influence water intake as well as health outcomes. Previous research by our group suggested sex may be a moderating factor but we did not find this in our analysis (already described in our manuscript).
- Alcohol intake can influence hydration physiology, as well as acting as a marker for lifestyle.
- Several other variables were included as markers of a healthy lifestyle (smoking, BMI, physical activity, processed meat, wholegrains, energy intake). Prior evidence suggests water intake and healthy lifestyle factors may correlate so we aimed to mitigate this effect.
- Since lifestyle can affect other markers of metabolic health, we controlled for such factors (ApoA, ApoB, hypertension, use of lipid lowering medication) to help isolate the impact of water *per se*.
- Education was included as a marker of socioeconomic status which potentially impacts lifestyle and health outcomes.

Reviewer #1 comment: Line 175 and Line 260 – change “waiter” to “water”

Author response: Thank you for highlighting this, we have amended the typos.

Reviewer #1 comment: Can you comment somewhere on the expected persistence (or changes) in the fluid intake and dietary behaviors over the course of the ~20 years until follow up?

Author response: It is currently unclear how exactly water intake is expected to change over a 20 year period in adults. Some factors, such as illness and obesity, have been reported to cause a change in drinking behaviours, and we adjusted for these where possible (e.g. BMI adjustment or removing incidence of diabetes). In addition, ageing is known to associated with lower fluid intake, but it is unclear whether this is a gradual linear decline or a more rapid decline followed by a plateau. <https://onlinelibrary.wiley.com/doi/epdf/10.1002/ajhb.23338?saml_referrer>

Some evidence suggests a reduction in water intake occurs after the age of 60 years, by approximately half a litre per day. Our median age was ~58 years at baseline, around the time when fluid intake may be expected to drop. We can only assume if this drop continues with older ages, it would be fairly homogenous.

<https://pubmed.ncbi.nlm.nih.gov/27139510/>

As such, we have included the following in our manuscript (lines 391-397):

“In addition, it is likely that drinking behaviours change over time, and we are unable to quantify this change in these analyses. Fluid intake typically decreases with ageing, particularly after 60 years [41]. Based on this, we can assume participants likely reduced their fluid intake during the study period, and assume this is roughly proportional between participants. However, further research should confirm or refute this, and our results should be taken within the context of this unknown.”

Reviewer #1 comment: Thank you for including a continuous model as well!

Reviewer #2 comment: The manuscript is not well written and in many sections is ambiguous.

Author response: We welcome suggestions for improvement.

Reviewer #2 comment: Cardiometabolic factors are mentioned in the title of the article, but only diabetes and CAD have been investigated.

Author response: Our title uses the term “cardiometabolic disease” which we felt encompassed both CAD and T2D, as two of the major cardiometabolic diseases. We would be happy to change the title if the reviewer has a suitable alternative.

Reviewer #2 comment: The final results of the study do not seem logical. According to the amount of water intake reported in the participants in table 2, the mechanism proposed in the discussion section is not correct (page 18, line 303). According to Table 1, the incidence of CAD was lower in subjects who received more water, while the final result of this study shows the opposite.

Author response: Thank you for highlighting this. The discrepancy is likely from adjustment, i.e. Table 1 is unadjusted and therefore reflects the influence of (e.g.) confounding factors.

Reviewer #2 comment: While appreciating the efforts of the authors, it seems that measurement error, out of date data, and the way of statistical analysis have caused disruption in the results.

Author response: We appreciate the limitations in our methods and hopefully do not portray these as definitive answers, rather added data to be considered within the wider context of the literature base, helping to build new testable hypotheses.

We hope we have adequately addressed comments.

Yours sincerely,

Harriet Carroll, PhD (on behalf of all authors)
