# Supplementary material for: The association between water intake and future cardiometabolic disease outcomes in the Malmö Diet and Cancer cardiovascular cohort
Source: PLoS One. 2024 Jan 19;19(1):e0296778. doi: 10.1371/journal.pone.0296778 (PMC10798487; doi:10.1371/journal.pone.0296778)
Supplement: Attachment — Submitted filename: Response to Reviewers.docx [file pone.0296778.s008.docx]

Dear Editor,

Thank you for arranging further review of our manuscript and please pass our thanks onto the reviewers for their thoughtful and helpful comments on the manuscript. We have addressed their additional comments below and submitted a tracked changes and clean manuscript.

**Reviewer comment:** Reviewer #1: Thank you for addressing my comments. Please note very minor grammatical error in line 60, I believe this should be "mediated". Otherwise, I appreciate you incorporating my suggestions into the manuscript and am happy to accept this manuscript.

**Author comment:** Thank you for your thoughtful comments. We have amended the error on line 60.

**Reviewer comment:** Reviewer #3: Thank you for your great study and I am sure you had to put a lot of efforts. In general, methods are too vague. More details are required.

Line 103-106: Precise information is required here, including how to process this data, how to assess plain and total water intake by these measurements, how to capture water intake outside of meals, how 1,2, and 3 methods used to calculate total water intake etc.

**Author comment**: Thank you for highlighting that this was unclear. To improve clarity, we have amended the section (lines 102-121) as follows (edits in red below):

“Dietary data were collected via a modified diet history method designed to capture both current and usual diet (described previously [26]), which included: (i) a 7-day food diary mainly covering lunch and dinner meals; (ii) a 168-item semi-quantitative diet history food frequency questionnaire (FFQ) of regularly eaten foods outside the main meals and not covered by the food diary; (iii) a 45-60 min diet history interview including questions about usual portion sizes, food preparation practices, and to check for overlap between the food diary and the FFQ. The validity of these methods has previously been reported [27]. These data were summarised and converted to nutrient intakes based upon the Swedish Food Database PC KOST2-93. The coding routines were slightly changed in Sept 1994 to shorten the interview time. The change did not have any major influence on the ranking on individuals on dietary intakes [26]. For the present analyses, water was defined in two different ways, with intakes split into tertiles to determine low, moderate, and high intakes for analyses:

1. Plain water: sum of mineral and tap water intake (average data intake recorded in the food diary)
2. Total water: reported average daily intake of all foods and beverages ([intake frequencies recorded in the 7-day food diary × portion sizes from the diary or diet history interview] + [intake frequencies × portion sizes from the FFQ]) was converted to water intake by multiplying with factors for water content obtained from PC KOST2-93 and summed”

**Reviewer comment:** Line 146: What is the justification to exclude participants who had a history of CAD or type 2 diabetes from analyses? This might change the results as the number of CAD and diabetes is intentionally decreased, and water intake may already (or may not) associate with them at baseline for some participants.

**Author comment:** We excluded those with current CAD or type 2 diabetes diagnoses so we could assess new-onset disease risk associated with water intake. We have clarified this in-text to read (lines 155-156, edits in red below):

“Participants who had a history of CAD or type 2 diabetes (meeting any diabetes criteria, as described above) at baseline (in order to assess the relationship with new-onset disease risk), or had missing data of exposures relevant to the analyses, were excluded from further analyses”

**Reviewer comment:** Line 154: what is the cut point of low, moderate, and high intake, and the citation is required here.

**Author comment:** As described on line 154, we split water intakes into tertiles in order to ascertain low, moderate, and high categories. We have added the cut-points found to the text (lines 162-164, edits in red below):

“…according to tertiles (median [interquartile range, IQR] plain water intakes: low: 199 [93, 298]; moderate: 564 [474, 662]; high: 1140 [945, 1506] mL/d) of plain water”

In addition, for improved clarity, we have added to lines 113-114 the following (edits in red below):

“For the present analyses, water was defined in two different ways, with intakes split into tertiles to determine low, moderate, and high intakes for analyses:…”

**Reviewer comment:** Line 182-188: Justifications and citations are required for each cut point for sensitivity.

**Author comment:** In addressing this comment, we realised we missed one of the sensitivity analyses conducted in the methods. As such “(ix) excluding diet changers” has been added (lines 205-206).

For sensitivity analysis (i) we have added a reference to Carroll *et al.* (2016) who used 240 mL cup sizes (line 192) and in the discussion (lines 352-363) we discussed that the rationale was in case categorisation skewed the results. Other sensitivity models are also discussed in the discussion section: Model (i) – lines 352-363; Model (iv) – lines 336-345, 361-363; Model (v) – lines 407-410; Model (vii) – lines 330-332; Model (ix) – lines 419-428.

However, we run these analyses to improve our confidence in the results and provide insight that might help explain our findings (hence the heavy focus in the discussion section). As such, we are not sure what references would be appropriate for this. We have however included our justification for each model in the methods, so it now reads (edits in red below, lines 191-206 in manuscript):

“Sensitivity analyses additionally analysed:

(i) plain and total water in cups (240 mL) per day (as per Carroll et al., 2016 [11]) (as categorisation can reduce power); (ii) model without reporter category adjustment (in case there was collinearity with other included variables such as BMI and as reporting accuracy could not be considered as a confounder as it does not influence disease development); (iii) plausible energy reporters only (to reduce the chance that those more likely reporting inaccurately were influencing results); (iv) removal of the top 10 % of fluid intakes (to account for potential illness-induced polydipsia and misreporters); (v) removal of those with type 2 diabetes/CAD within 2 years of baseline measurements (in case recent awareness of ill-health/diagnosis altered health behaviours); (vi) inclusion of hypertensive participants only (to see whether pathology could be influencing results); (vii) inclusion of normotensive participants only (to see whether pathology could be influencing results); (viii) energy adjusted dietary variables (to ensure results were not skewed by relative intake levels); (ix) excluding diet changers (to remove the influence of known dietary changes which could add noise to the data).”

**Reviewer comment:** Line 190: When is referring to follow-up time?

**Author comment:** We have amended the text to be clear that this was in reference to disease onset time (line 208, edit in red below):

Mean (± standard deviation) follow-up time to incident diagnosis was 20 ± 7 (median 23, IQR 17, 25) years for CAD, and 20 ± 7 (median 23, IQR 15, 25) years for type 2 diabetes

**Reviewer comment:** Line 194-196: Even 1140 g/d can be categorized as “low drinker” in some previous studies, so these cut points might not capture results appropriately. At least, it is not appropriate to call “high” at this level.

**Author comment:** We agree that intakes can be variable depending on the study and aims. In order to provide a fairer comparison, we wanted to have even comparator groups, hence we opted for tertiles of intake (hopefully clarified in a previous comment above). Since plain water intake was approximately 1 L/d different between the low and high groups, we hopefully sufficiently captured adequate differences. Nonetheless, we have added to the discussion (lines 398-402) the following:

“In addition, whilst splitting water intakes into tertiles prevented issues such as sample size differences skewing the results, and whilst we believe we captured sufficient differences in plain water intake between low (199 mL/d) and high (1140 mL/d) intakes, it could be argued that the high intake group still were not consuming high enough intakes to induce meaningful physiological changes.”

**Reviewer comment:** Table 1 &2: Need to reorganize as it is hard to see.

**Author comment:** Apologies for this. We have changed the page orientation to allow for a wider table which is hopefully clearer to read. However if you have more specific suggestions for improved clarity, we would be happy to take those on board.

**Reviewer comment:** Discussion needs to be changed accordingly.

**Author comment:** We hope we have addressed your points and amended the discussion appropriately.

**Reviewer comment:** Reviewer #4: Thank you for the opportunity to review the current manuscript regarding the relationship between recorded water intake and longitudinal risk for development of T2DM and CAD within a population of the Malmo Diet and Cancer Cohort. There are a few questions regarding the statistical approach that I hope the authors can provide clarity for.

It appears that the collinearity between the data used to group participants into tertiles (i.e., plain water or total water) and the covariate of model 5 (plain water) would limit the ability of the grouping to account for any other variation in the dependent variables (i.e., T2DM or CAD). Additionally, the description of the "plain water only" variable as total water minus plain water is confusing because by that math, the difference would be every source of water intake except plain water. If the covariate was indeed "other water sources" that could answer the question if T2DM or CAD risk differs by plain water tertile, however it does not address the underlying physiological question/hypothesis related to water intake and disease.

**Author comment:** The rationale for this inclusion was to remove collinearity with plain water as the independent variable (i.e. total water minus plain water—which is another way of saying “other water sources” as you describe). To check this did not affect results, we re-ran the model without this variable, with almost identical results (see table below):

|  | HR | 95 % CI | *p* |
| --- | --- | --- | --- |
|  | Plain water | | |
|  | Type 2 diabetes | | |
|  | Moderate (*versus* low) intake | | |
| Original model 5 | 1.05 | 0.97, 1.13 | 0.202 |
| Without “total minus plain water” variable | 1.05 | 0.98, 1.13 | 0.196 |
|  | High (*versus* low) intake | | |
| Original model 5 | 1.07 | 0.99, 1.16 | 0.075 |
| Without “total minus plain water” variable | 1.07 | 0.99, 1.16 | 0.072 |
|  | Coronary artery disease | | |
|  | Moderate (*versus* low) intake | | |
| Original model 5 | 0.99 | 0.93, 1.08 | 0.993 |
| Without “total minus plain water” variable | 1.02 | 0.94, 1.10 | 0.696 |
|  | High (*versus* low) intake | | |
| Original model 5 | 1.12 | 1.03, 1.21 | 0.008 |
| Without “total minus plain water” variable | 1.13 | 1.04, 1.22 | 0.005 |

Please note that in addressing this comment, we noticed the HR, CI, and *p*-values were incorrect for the plain water CAD model. The corrections are outlined below and have been amended where appropriate in text, tables, and supplementary material. Because of this error, we checked all other results, and found two more typos, also outlined below. Thank you for this comment which triggered these checks. Luckily none of the typos affected the interpretation of the results.

| Model | Original | Correction |
| --- | --- | --- |
| CAD plain water model 5, moderate vs low intake | HR 0.99  95 % CI 0.93, 1.08  p = 0.993 | HR 1.02  95 % CI 0.94, 1.10  p = 0.662 |
| CAD plain water model 5, high vs low intake | HR 1.12  95 % CI 01.03, 1.21  p = 0.008 | HR 1.13  95 % CI 1.04, 1.23  p = 0.004 |
| T2D plain water model 2, moderate vs low intake | HR 1.07  95 % CI 0.99, 1.24  p = 0.090 | HR *No change*  95 % CI 0.99, 1.15  p = *No change* |
| T2D total water model 1, high vs low intake | HR 1.10  95 % CI 1.02, 1.19  p = 0.032 | HR *No change*  95 % CI *No change*  p = 0.011 |

**Reviewer comment:** The authors mention that the purpose of converting a continuous variable into a categorical variable was done so to reduce the impacts of outliers. However, the concerns of such practice remain as outlined in the following reference, Howard Wainer, Marc Gessaroli & Monica Verdi (2006) Visual Revelations, CHANCE, 19:1, 49-52, DOI: 10.1080/09332480.2006.10722771

There is also mention of a continuous model on line 338 of the Discussion, however I am not able to locate this analysis within the methods or results section.

**Author comment:** We ran a continuous model as a sensitivity analysis for exactly the reason you describe. This is reported in Supplementary Material Table S1. For ease, we have copied the relevant sections of the table below. Hopefully we have adequately addressed the differences in the discussion (lines 352-363). Whilst there are some differences, it seems possible that categorisation has allowed for us to see that the differences are between high *versus* low intakes, rather than a linear association as a continuous model might suggest. We have made this point clearer in the discussion (lines 353-356), which now reads (edits in red below):

“For plain water, both type 2 diabetes and CAD were associated with 2 % higher risk in continuous models, which is discordant with both our original analyses (with null associations between moderate versus low intakes), though suggests the relationship may not be linear as the results are in line with the comparisons between high versus low intakes for CAD.”

|  | HR | 95 % CI | *p* | HR | 95 % CI | *p* |
| --- | --- | --- | --- | --- | --- | --- |
|  | PLAIN WATER | | | | | |
|  | TYPE 2 DIABETES | | | CORONARY ARTERY DISEASE | | |
| Main analysis: Moderate versus low intake | **1.05** | **0.97, 1.13** | **0.202** | **0.99** | **0.93, 1.08** | **0.993** |
| Main analysis: high versus low intake | **1.07** | **0.99, 1.16** | **0.075** | **1.12** | **1.03, 1.21** | **0.008** |
| Continuous model | 1.02 | 1.00, 1.01 | 0.012 | 1.02 | 1.01, 1.04 | 0.003 |
|  | TOTAL WATER | | | | | |
| Main analysis: Moderate versus low intake | **1.04** | **0.96, 1.12** | **0.301** | **1.04** | **0.96 1.13** | **0.335** |
| Main analysis: high versus low intake | **1.07** | **0.99, 1.16** | **0.107** | **1.17** | **1.07, 1.27** | **< 0.001** |
| Continuous model | 1.01 | 1.00, 1.02 | 0.227 | 1.02 | 1.01, 1.03 | < 0.001 |

**Reviewer comment:** The results of this investigation are compelling as they do not agree with previous research in the area. The authors have devoted considerable space within the current manuscript to controls and checks which should enhance the readers confidence in the outcomes. Addressing the two concerns above will provide further confidence for the reader when reading something they may not have previously believed to be the case and avoid the misappropriated sentiment of "Well, this is just a case of fancy p hacking."

**Author comment:** Thank you for your thoughtful and helpful comments. Hopefully we have addressed these and indeed they improve confidence to readers.

We hope we have adequately addressed all comments.

Kind regards,

Harriet Carroll, on behalf of all authors
